# CT-based radiomics features in the prediction of thyroid cartilage invasion from laryngeal and hypopharyngeal squamous cell carcinoma

**DOI:** 10.1186/s40644-020-00359-2

**Published:** 2020-11-11

**Authors:** Ran Guo, Jian Guo, Lichen Zhang, Xiaoxia Qu, Shuangfeng Dai, Ruchen Peng, Vincent F. H. Chong, Junfang Xian

**Affiliations:** 1 Department of Radiology, Beijing Tongren Hospital, Capital Medical University, No 1 Dongjiaominxiang, Dongcheng District, Beijing, 100730 China; 2 Department of Radiology, Beijing Luhe Hospital, Capital Medical University, No 82 Xinhua South Road, Tongzhou District, Beijing, 101149 China; 3 Huiying Medical Technology Co., Ltd, Beijing, 100000 China; 4 Department of Diagnostic Imaging, National University Health System, Singapore, 119074 Singapore

**Keywords:** Radiomics, Larynx, Hypopharynx, Squamous cell carcinoma, Thyroid cartilage

## Abstract

**Background:**

Laryngeal and hypopharyngeal squamous cell carcinoma (LHSCC) with thyroid cartilage invasion are considered T4 and need total laryngectomy. However, the accuracy of preoperative diagnosis of thyroid cartilage invasion remains lower. Therefore, the purpose of this study was to assess the potential of computed tomography (CT)-based radiomics features in the prediction of thyroid cartilage invasion from LHSCC.

**Methods:**

A total of 265 patients with pathologically proven LHSCC were enrolled in this retrospective study (86 with thyroid cartilage invasion and 179 without invasion). Two head and neck radiologists evaluated the thyroid cartilage invasion on CT images. Radiomics features were extracted from venous phase contrast-enhanced CT images. The least absolute shrinkage and selection operator (LASSO) and logistic regression (LR) method were used for dimension reduction and model construction. In addition, the support vector machine-based synthetic minority oversampling (SVMSMOTE) algorithm was adopted to balance the dataset and a new LR-SVMSMOTE model was constructed. The performance of the radiologist and the two models were evaluated with receiver operating characteristic (ROC) curves and compared using the DeLong test.

**Results:**

The areas under the ROC curves (AUCs) in the prediction of thyroid cartilage invasion from LHSCC for the LR-SVMSMOTE model, LR model, and radiologist were 0.905 [95% confidence interval (CI): 0.863 to 0.937)], 0.876 (95%CI: 0.830 to 0.913), and 0.721 (95%CI: 0.663–0.774), respectively. The AUCs of both models were higher than that of the radiologist assessment (all *P* < 0.001). There was no significant difference in predictive performance between the LR-SVMSMOTE and LR models (*P* = 0.05).

**Conclusions:**

Models based on CT radiomic features can improve the accuracy of predicting thyroid cartilage invasion from LHSCC and provide a new potentially noninvasive method for preoperative prediction of thyroid cartilage invasion from LHSCC.

**Supplementary Information:**

The online version contains supplementary material available at 10.1186/s40644-020-00359-2.

## Background

Laryngeal and hypopharyngeal squamous cell carcinoma (LHSCC) are common malignant tumors in the head and neck [1, 2]. The International Agency for Research on Cancer estimated that 177,422 and 80,608 new cases of laryngeal carcinoma and hypopharyngeal carcinoma would be diagnosed in 2018 globally, directly accounting for 94,771 and 34,984 deaths, respectively, and approximately 12 to 43% of patients are predicted to be diagnosed with cartilage invasion during the diagnosis of LHSCC [3, 4]. Over-staging of thyroid cartilage invasion results in unnecessary total laryngectomy, whereas underestimation results in a higher risk of local residual tumor and recurrence [5–7]. Therefore, accurate evaluation of thyroid cartilage invasion in patients with LHSCC is crucial for preoperative TNM staging and treatment [1, 2, 4, 5, 8].

At present, conventional imaging modalities, for example computed tomography (CT) and magnetic resonance imaging (MRI), play an essential role in the diagnosis of thyroid cartilage invasion [9]. According to the literature, the sensitivity of conventional CT in the diagnosis of thyroid cartilage invasion is low (49–71%) because of the great variability of ossification in the thyroid cartilage [10, 11]. The introduction of dual-energy CT has improved the sensitivity of CT to 89% [10, 12]. However, dual-energy or spectral CT is expensive and not all hospitals can afford the technology. The reported sensitivity of MRI is 64 to 96% and the specificity is relatively low (64–75%) [2, 10, 13, 14]. Furthermore, inflammatory changes in the thyroid cartilage can be mistaken for tumors [11]. Conventional imaging diagnosis is often based on the qualitative analysis of radiologists and has limitations in the assessment of thyroid cartilage invasion.

Radiomics is a quantitative analysis method based on medical images and uses a large number of algorithms to transform the region of interest (ROI) in medical images into high-dimensional features [15]. It can be used to analyze the heterogeneity of an entire tumor based on hundreds of quantitative features and also analyze the relationship between the biological and imaging characteristics of the tumor quantitatively [15–17]. It is widely used in research on tumor diagnosis, prognosis, and the prediction of treatment response [17–21]. To the best of our knowledge, there is no study in the literature that has evaluated the application of CT radiomics for the prediction of thyroid cartilage invasion of LHSCC. In addition, a balanced dataset is of great importance in the creation of a good training set [22, 23]. In 2002, Chawla et al. [24] proposed the classic synthetic minority oversampling technique (SMOTE), which over-sampled minority classes by generating “synthetic” examples to balance the dataset. The SMOTE technique in combination with support vector machine [(SVM), SVMSMOTE] can further improve the learning ability of classifiers [25–27].

The purpose of our study is to assess the value of radiomics features with and without the SVMSMOTE to predict thyroid cartilage invasion in LHSCC based on CT images.

## Materials and methods

### Patients

**Fig. 1:**
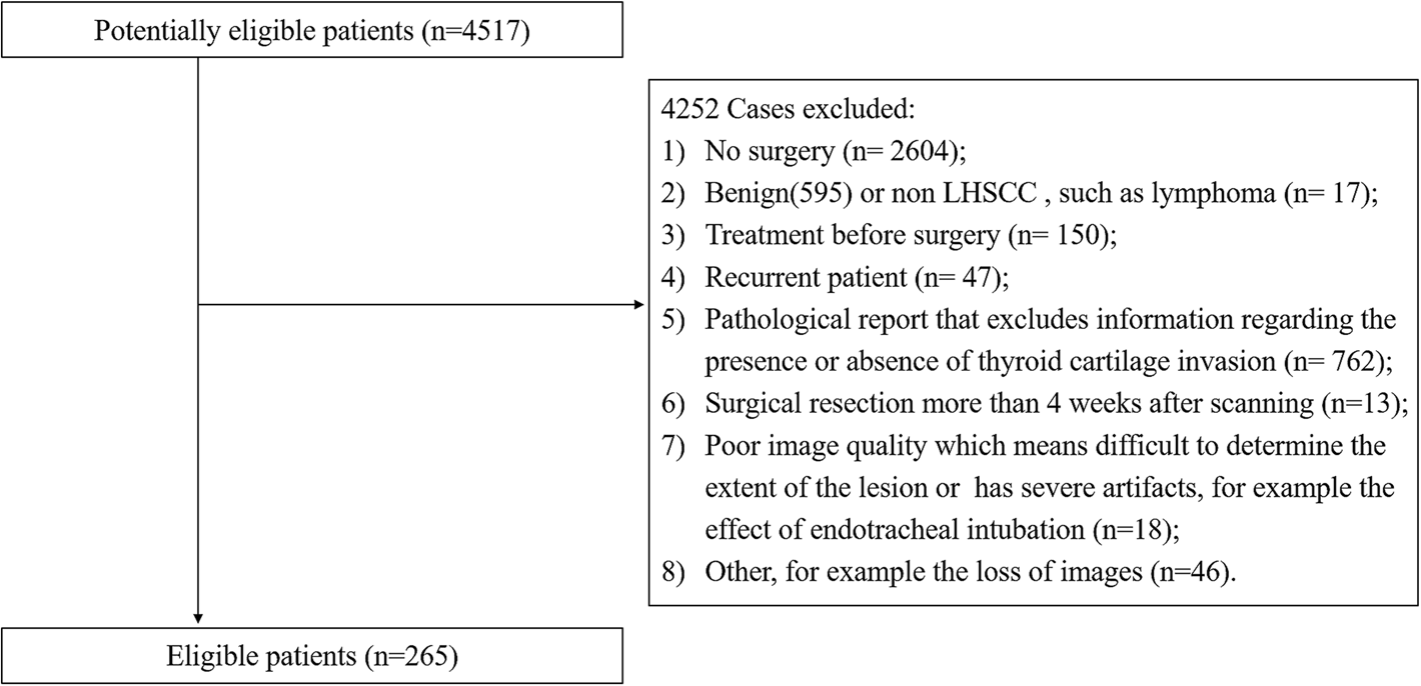
Patient recruitment pathway

Our institutional review board approved this retrospective study. The study population consisted of patients who had preoperative contrast-enhanced CT (CE-CT) examination for suspected hypopharyngeal and laryngeal masses (from January 2009 to November 2017). The inclusion criteria were as follows: 1) all patients were confirmed by histopathology; 2) no preoperative treatment; 3) surgical resection within 4 weeks after scanning; and 4) excellent image quality clearly showing the extent of the lesions. The exclusion criteria were as follows: 1) no surgery; 2) benign or non-LHSCC patients; 3) treatment before surgery; 4) recurrence; 5) pathological report that excluded information regarding the presence or absence of thyroid cartilage invasion; 6) image quality that is too poor to determine the extent of the lesion or has severe artifacts. The details of the patient recruitment pathway are shown in Fig. 1. Ultimately, 265 patients were enrolled in this study and were divided into two groups: 1) LHSCC patients with thyroid cartilage invasion (86); 2) LHSCC patients without thyroid cartilage invasion (179).

### CT imaging acquisition and processing

CE-CT images were obtained using three scanners: the SOMATOM Definition Flash CT scanner (Siemens Healthcare, Germany) and the Brilliance 64 and iCT 256 multi-detector row CT (MD-CT) scanners (Philips Medical Systems, Nederland B.V.). Before scanning, the patients’ heads were fixed and they were instructed to keep their head and neck still, breathe calmly and avoid swallowing. The scanning parameters were as follows:

SOMATOM Definition Flash CT scanner: tube voltage, 80/Sn140 kVp; reference tube current of 100 mAs and 50 mAs, respectively, with automatic tube current modulation (Care Dose 4D); detector collimation, 128 × 0.6 mm; pitch, 1.0; field of view, 200 ~ 225 mm; pixel size, 512 × 512; rotation time, 0.28 s.

Philips Brilliance 64-MDCT scanner: tube voltage, 120 kVp; automatic tube current modulation (ATCM); detector collimation, 64 × 0.625 mm; pitch, 0.8; field of view, 200 ~ 225 mm; pixel size, 512 × 512; rotation time, 1.0 s.

Philips Brilliance iCT 256-MDCT scanner: tube voltage, 120 kVp; ATCM; detector collimation, 128 × 0.625 mm; pitch, 0.8; field of view, 200 ~ 225 mm; pixel size, 512 × 512; rotation time, 0.75 s.

Other parameters were: slice interval, 1 mm; slice thickness, 1 mm; reconstructed section thickness, 3 mm; slice interval, 3 mm.

The patients were positioned in a supine position. The scanning region was from the skull base to the thoracic inlet. Contrast agent was injected into the anterior elbow vein or dorsal hand vein at a rate of 3 ml/s with an injection dose of 1 ml/kg. CT scans were acquired at 50 s (Brilliance 64 and iCT 256 MDCT scanners) or 70 s (SOMATOM Definition Flash CT scanner) after the administration of iodine contrast agent (iopaconol, 300 mg/ml iodine, Shanghai Xinyi Pharmaceutical Co., Ltd., China).

### Radiologist assessment of the thyroid cartilage invasion

**Fig. 2:**
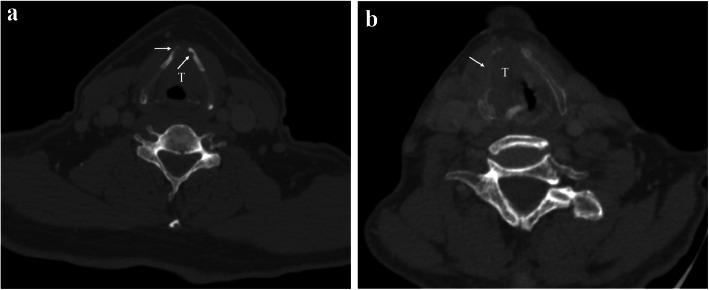
**a** Axial CE-CT image. Histopathology confirmed thyroid cartilage invasion in a 57-year-old man with supraglottic laryngeal carcinoma. Thyroid cartilage shows focal erosion (white arrow) that involves the inner cortex but do not penetrate the outer cortex, which is defined as minor invasion. **b** Axial CE-CT image for a 61-year-old man depicts a large tumor at the level of the glottic region that penetrates the right thyroid cartilage and presents as an extralaryngeal mass (white arrow) and thyroid cartilage is lysis, which was defined as major invasion

Two radiologists (R.G. and L.C.Z with 5 and 3 years of head-neck radiologic experience, respectively), who were blinded to the patients’ clinical and pathological information, interpreted all CT images to assess thyroid cartilage involvement. The following criteria were considered to be thyroid cartilage invasion: 1) minor cartilage erosion or lysis, which was defined as tumor invasion of the inner cortex that did not penetrate the outer cortex; 2) major cartilage lysis or penetration, which was defined as tumor penetration of the outer cortex of the cartilage or extralaryngeal soft tissue [6, 12, 28] as is shown in Fig. 2.

### Radiomics assessment of the thyroid cartilage invasion

#### Tumor segmentation

**Fig. 3:**
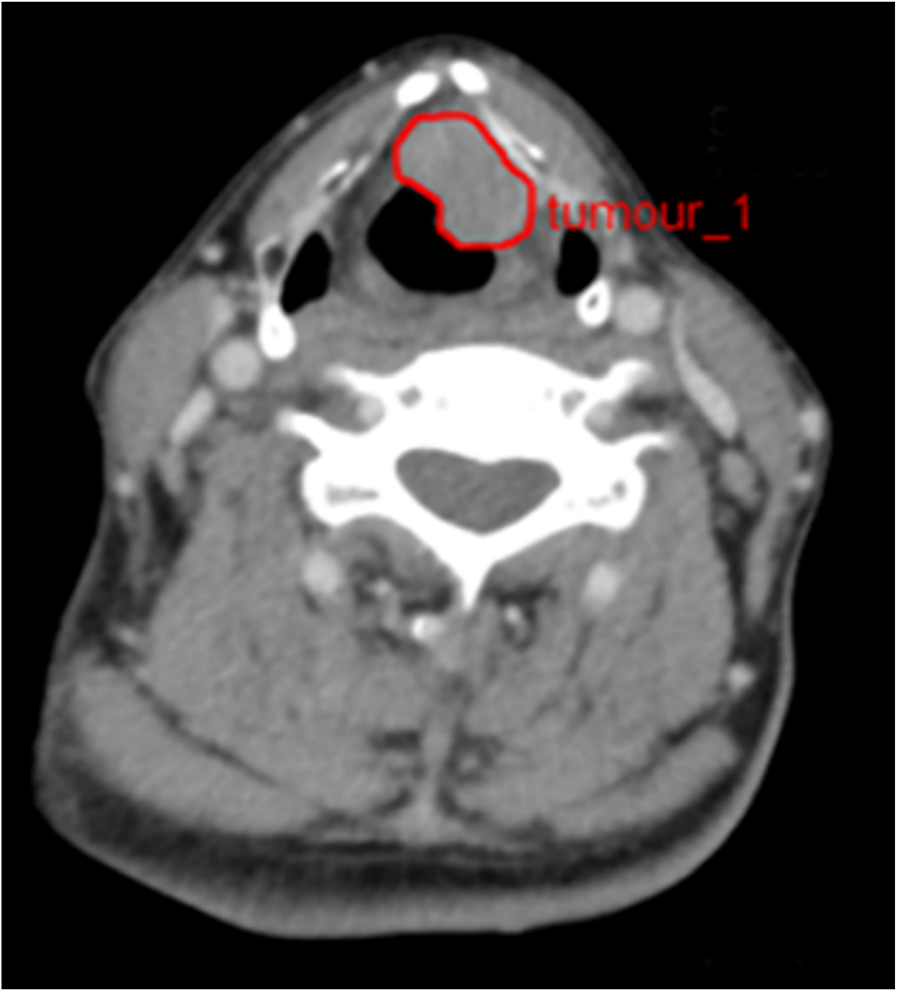
An example of manual segmentation in CE-CT image from a 65-year-old male patient with supraglottic laryngeal carcinoma. Red contour was drawn to contain the whole tumor region in one slice

The volumes of interest (VOI) containing the entire tumor for each patient were contoured on all slices by two radiologists (R.G. as reader 1 and L.C.Z. as reader 2). The guidelines used for contouring were as follows: 1) To avoid partial volume effects, the outlines were delineated slightly within the borders of the tumor on each slice and no ROI was delineated on the first and last slices where the lesion was visible; 2) cystic areas were avoided; 3) for thyroid cartilage involvement, the area where the tumor involved the thyroid cartilage was delineated and if the tumor across the cartilage to form an extralaryngeal mass, the extralaryngeal area was also delineated, but cartilage that appeared normal on the CT was avoided; 4) the extent of the lesion was carefully determined by adjusting the window width and level and performing multi-planar reconstruction. Reader 1 delineated the VOI for all patients manually, while reader 2 delineated the VOI for 50 patients selected randomly from the cohort. The inter-class correlation coefficient (ICC) among 1029 features was calculated for the latter 50 patients. Reader 3 (J.G), a senior radiologist with 15 years of relevant experience, examined each VOI during the process of tumor segmentation. When drawing or checking the VOI, the three readers were blinded to the information for each patient. An example of the manual segmentation is shown in Fig. 3.

#### Radiomics feature extraction

A total of 1029 radiomics features were extracted for each patient from the original and filtered CE-CT images based on the VOI, including intensity histogram features, shape and size features, and texture features. The filters consisted of an exponential filter, square filter, square root filter, logarithmic filter, and wavelet decomposition. The texture features were further divided into three subgroups: gray level co-occurrence matrix (GLCM), gray level run length matrix (GLRLM), and gray level size zone matrix (GLSZM). The definitions and names of the radiomics features were in accordance with the Imaging Biomarker Standardization Initiative (IBSI) [29] and consistent with the studies by Shu et al. [20] and Liang et al. [30]. Details of the radiomics features are shown in Supplementary S1.

#### Feature standardization and selection

Before feature selection, each radiomics feature was standardized in order to eliminate bias from the value ranges for different features. The Kruskal-Wallis nonparametric test (KW test) [31] was used to remove the features showing significant statistical differences among the three scanners, and the remaining features were selected using the least absolute shrinkage and selection operator regression (LASSO) method [32].

The process of LASSO-feature selection in our study was as follows: Firstly, the optimal coefficient of regularization α was found via the minimum average mean square error among a set of candidate values using ten-fold cross validation. Secondly, features with non-zero coefficients in the LASSO method were selected using the whole dataset based on the optimal α. Thirdly, the remaining features were further selected based on the absolute values of coefficients that were greater than 0.04 in the LASSO method to avoid over-fitting and improve the generalization of classifiers.

Furthermore, to reduce the impact of a dataset imbalance on the prediction model, the SVMSMOTE technique was implemented to generate pseudo-data for patients with invasion based on selected features using the KW test to reach a one-to-one distribution ratio between the two groups. Afterward, the dataset containing pseudo-data was subjected to the above LASSO-feature selection process.

#### Statistical analysis

The interobserver reproducibility was assessed based on the intraclass correlation coefficients (ICCs). The Student’s *t* test and the Chi-square test were used to compare the general characteristics of the patients in the two groups. The diagnostic performance of the radiologist was evaluated using a receiver operating characteristics (ROC) curve with the calculated area under the curve (AUC). The following metrics were calculated: sensitivity, specificity, accuracy, precision, F1-score, Cohen’s kappa coefficient (Kappa), and Matthews correlation coefficient (MCC). The MCC was calculated with the equation

$$ \left[\left(\mathrm{TP}\times \mathrm{TN}\right)-\left(\mathrm{FP}\times \mathrm{FN}\right)\right]/\sqrt{\left(\mathrm{TP}+\mathrm{FP}\right)\left(\mathrm{TP}+\mathrm{FN}\right)\left(\mathrm{TN}+\mathrm{FP}\right)\left(\mathrm{TN}+\mathrm{FN}\right)} $$

Where TP, FP, TN, and FN refer to the true positive, false positive, true negative, and false negative values, respectively.

Two CE-CT radiomics models were used to predict thyroid cartilage invasion from LHSCC. The models were constructed using logistic regression (LR) based on the two feature sets described in the Feature standardization and selection section (the LR model using the features selected by LASSO and the LR-SVMSMOTE model using the features selected by LASSO and SVMSMOTE) with five-fold cross validation.

In five-fold cross validation, the whole dataset was randomly partitioned into five equal sized subsets. A single subset was retained as the validation dataset and the remaining k-1 subsets were used to create the training dataset. The cross-validation process was repeated five times, with each of the subsets used once as the validation dataset.

The predictive performance of both models was evaluated using a ROC curve and statistical metrics mentioned above. The ROC curves were compared with the Delong test [33]. The tests in our study were two-tailed, and a *P*-value less than 0.05 was considered to indicate statistical significance.

### Platform and packages

Manual tumor segmentation and feature extraction were both performed with the Radcloud platform (https://mics.radcloud.cn, Huiying Medical Technology Co., Ltd). Feature standardization, selection, and statistical analysis were performed with the Anaconda3 platform (https://www.anaconda.com/) using the Python 3.6 programming language (https://www.python.org/), mainly with the packages ‘scikit-learn’ (https://scikit-learn.org/) and ‘matplotlib’ (http://matplotlib.org/).

## Results

### General characteristics of the patients

In our study, 265 (253 men and 12 women; mean age, 60.4 ± 7.6) patients were enrolled, among which 86 (32.5%) with thyroid cartilage invasion and 179 (67.5%) without thyroid cartilage invasion. There were no significant differences in age, gender, and N stage between the two groups (*P* > 0.05). There were significant differences in the primary site (supraglottis, glottis, subglottis, hypopharynx) and T stage of the lesions between the two groups (*P* < 0.05). The general characteristics and tumor staging for the two groups are shown in Table 1.

**Table 1:** Patient general characteristics and tumor staging

General characteristics	With thyroid cartilage invasion	Without thyroid cartilage invasion	*P* Value
Number	86	179	
Age(mean ± SD, years)	59.7 ± 10.5	59.3 ± 9.4	0.263^a^
Gender			
male	80 (93.0%)	173 (3.4%)	0.184^b^
female	6 (7.0%)	6 (2.8%)	
Primary site			
Supraglottis	30 (34.9%)	82 (45.8%)	0.029^b^
Glottis	49 (57.0%)	69 (38.5%)	
Subglottis	1 (1.1%)	8 (4.5%)	
Hypopharynx	6 (7.0%)	20 (11.2%)	
T stage			
T1	0 (0)	25 (14.0%)	< 0.001^b^
T2	0 (0)	67 (37.4%)	
T3	44 (51.2%)	87 (48.6%)	
T4	42 (48.8%)	0 (0)	
N stage			
N0	60 (69.7%)	117 (65.4%)	0.717^b^
N1	14 (16.3%)	28 (15.6%)	
N2	12 (14.0%)	33 (18.4%)	
N3	0 (0)	1 (0.6%)	

*SD* standard deviation

*P* value < 0.05 is considered as a significant difference

^a^Student’s t test

^b^Chi-square test

### Interobserver reproducibility of radiologist assessment and radiomics

The evaluation of thyroid cartilage invasion by reader 1 and reader 2 showed good interobserver agreement, with an ICC of 0.803[95% Confidence Interval (CI):0.755 to 0.842]. Of the 1029 radiomics features, 877 were demonstrated to have good interobserver agreement, with ICCs from 0.750 to 0.999. Subsequent processes were based on the results reported by reader 1, who had longer work experience than reader 2.

### Diagnostic performance of radiologist assessment

Histopathologic diagnosis was used as the gold standard. Data used in the detection of thyroid cartilage invasion for LHSCC on CT versus histopathologic examination is shown in Table 2. The AUC, sensitivity, specificity, accuracy, precision, F1-score, Kappa, and MCC were 0.721(95%CI: 0.663–0.774), 74.4, 69.8, 71.3, 54.2, 0.627, 0.404, and 0.417%, respectively. The diagnostic performance of radiologist assessment in the detection of thyroid cartilage invasion is summarized in Table 4.

**Table 2:** Cross tabulation of thyroid cartilage invasion for LHSCC on CT versus histopathologic examination (cases)

CT diagnosis	Histopathologic diagnosis		Total
	With thyroid cartilage invasion	Without thyroid cartilage invasion	
With thyroid cartilage invasion	64	54	118
Without thyroid cartilage invasion	22	125	147
Total	86	179	265

*LHSCC* Laryngeal and hypopharyngeal squamous cell carcinoma

### Predictive performance of radiomics features

#### Radiomics feature selection

After the KW nonparametric test, there remained 740 features showing no significant differences among the three scanners in the original radiomics feature set. Twenty-two of these features were selected by the LASSO method. After pseudo-data generation with SVMSMOTE based on 740 features, 87 non-zero features were obtained with the LASSO method and 30 features were further selected because the absolute values of their coefficients in the LASSO method were greater than 0.04. The feature sets selected for the two models are shown in Table 3.

**Table 3:** The selected feature sets with LASSO and LASSO with SVMSMOTE

	Selected Features
LASSO (*n* = 22)	original_shape_LeastAxis
	original_shape_Elongation
	original_shape_Flatness
	logarithm_firstorder_Kurtosis
	logarithm_glrlm_HighGrayLevelRunEmphasis
	square_firstorder_10Percentile
	square_glrlm_ShortRunHighGrayLevelEmphasis
	exponential_glcm_Imc1
	exponential_glrlm_LongRunEmphasis
	exponential_glrlm_LongRunLowGrayLevelEmphasis
	wavelet-LHL_firstorder_Skewness
	wavelet-LHH_glcm_ClusterShade
	wavelet-HLL_firstorder_Energy
	wavelet-LLH_firstorder_Kurtosis
	wavelet-LLH_glcm_ClusterProminence
	wavelet-HHH_glszm_GrayLevelNonUniformity
	wavelet-HHH_glszm_LowGrayLevelZoneEmphasis
	wavelet-HHH_glszm_SmallAreaLowGrayLevelEmphasis
	wavelet-HHL_firstorder_Skewness
	wavelet-HHL_glrlm_ShortRunLowGrayLevelEmphasis
	wavelet-LLL_firstorder_Kurtosis
	wavelet-LLL_glcm_Correlation
LASSO with SVMSMOTE (*n* = 30)	wavelet-HLL_firstorder_Energy
	exponential_glrlm_LongRunEmphasis
	wavelet-HHL_firstorder_Skewness
	wavelet-HHL_glrlm_ShortRunLowGrayLevelEmphasis
	wavelet-HLH_glrlm_RunPercentage
	wavelet-LHL_firstorder_90Percentile
	square_glrlm_LongRunEmphasis
	logarithm_glrlm_ShortRunHighGrayLevelEmphasis
	wavelet-LHL_glcm_MaximumProbability
	wavelet-LLH_glszm_GrayLevelNonUniformity
	wavelet-LHL_firstorder_Mean
	wavelet-LHH_glcm_ClusterShade
	wavelet-LHL_glszm_ZonePercentage
	wavelet-HHL_glszm_GrayLevelVariance
	original_shape_Elongation
	wavelet-LLH_firstorder_10Percentile
	square_glcm_Correlation
	original_shape_Flatness
	wavelet-HHH_glszm_LowGrayLevelZoneEmphasis
	wavelet-LLL_glcm_Correlation
	exponential_glrlm_ShortRunHighGrayLevelEmphasis
	wavelet-LLL_firstorder_Kurtosis
	wavelet-LHL_firstorder_InterquartileRange
	wavelet-LLH_glcm_Contrast
	wavelet-LLH_firstorder_Energy
	wavelet-LLH_firstorder_Minimum
	wavelet-LHL_glszm_ZoneEntropy
	wavelet-HHH_glszm_GrayLevelNonUniformity
	original_glszm_ZoneEntropy
	original_shape_LeastAxis

Each feature is denoted as Filter_FeatureGroup_FeatureName and ‘Original’ indicates the radiomics features extracted from the original images without preprocessing

#### Predictive performance of two models

**Fig. 4:**
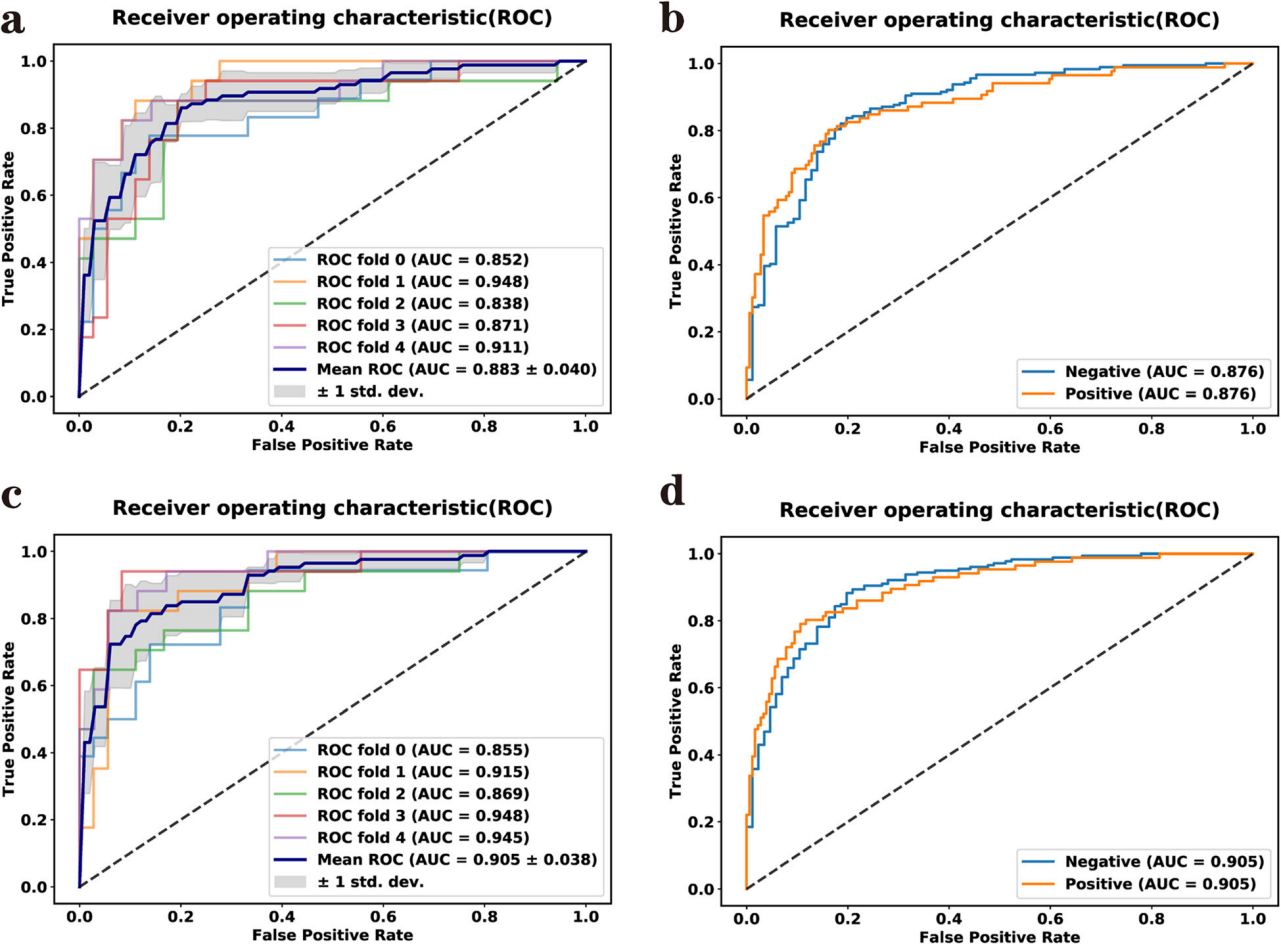
**a-b** ROC curve for the LR model. **c-d** ROC curve for the LR-SVMSMOTE model

**Fig. 5:**
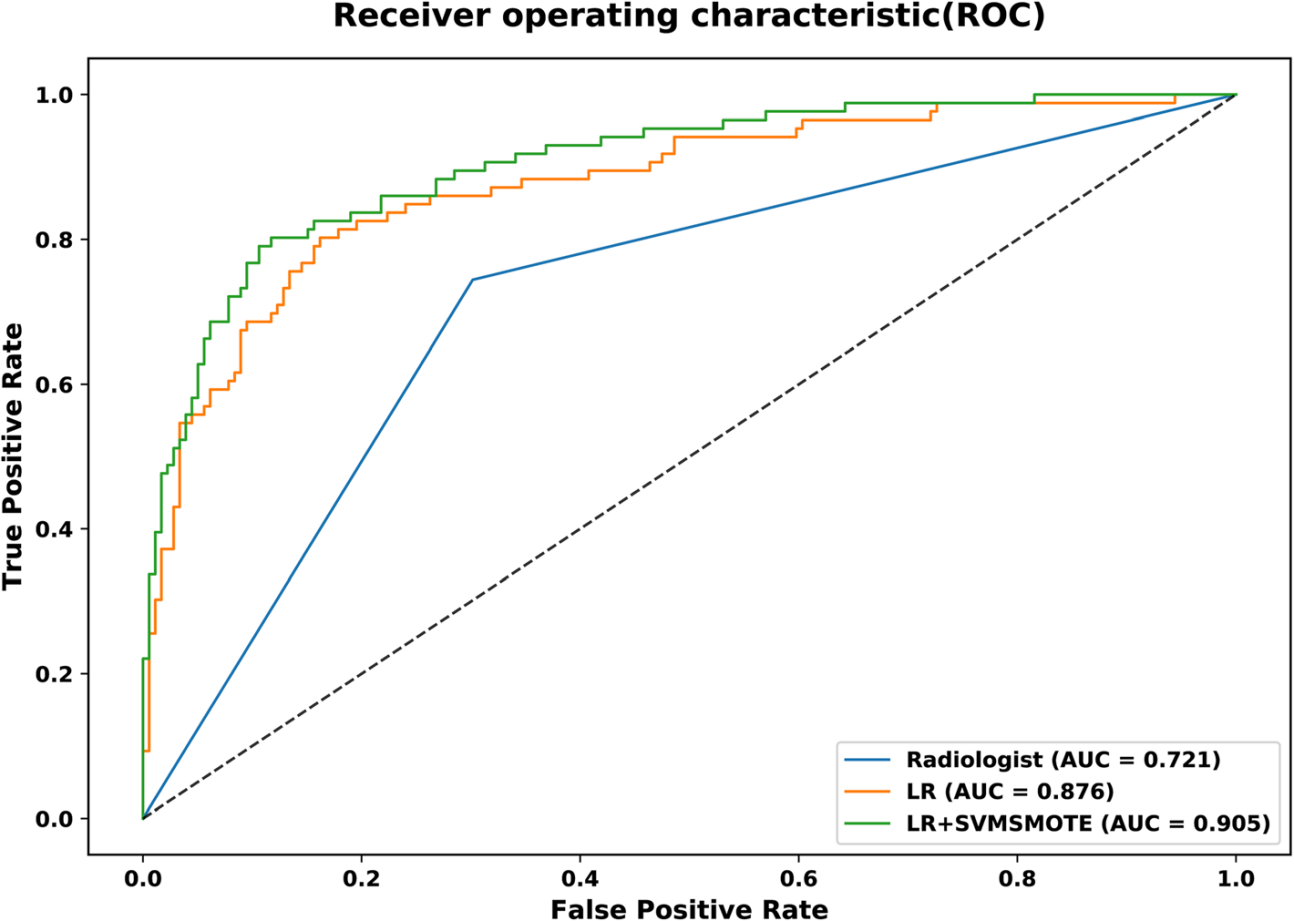
ROC curves for LR (orange) and LR-SVMSMOTE (green) models and radiologist assessment(blue) for the whole dataset to predict thyroid cartilage invasion from LHSCC

Figure 4a-b and c-d show the ROC curves for the LR model and the LR-SVMSMOTE model, respectively. The left image (Fig. 4a and c) show the mean result for the model with five-fold cross-validation and the right image (Fig. 4b and d) show the combined five-fold cross-validation results, respectively. Figure 5 shows that the models based on CT-radiomics predicted thyroid cartilage invasion of LHSCC with high AUC.

The predictive performance for each model is summarized in Table 4. The AUC, sensitivity, specificity, accuracy, precision, F1-score, Kappa, and MCC of the radiomics features were 0.876(95%CI: 0.830 to 0.913), 80.2, 83.8, 82.6, 70.4, 0.750, 0.618, and 0.621% for the LR model, respectively, and 0.905(95%CI: 0.863 to 0.937), 80.2, 88.3, 85.7, 76.7, 0.784, 0.677, and 0.677% for the LR-SVMSMOTE model, respectively. The LR-SVMSMOTE model had better AUC, but the improvement was not significant according to the Delong test (shown in Fig. 5, *P* = 0.050). The AUCs of the LR-SVMSMOTE model and LR model were higher than that of the radiologist assessment in the prediction of thyroid cartilage invasion from LHSCC (shown in Fig. 5, *P* < 0.001 for all).

**Table 4:** The diagnostic performance of radiologist assessment, LR model, and LR-SVMSMOTE model in the prediction of thyroid cartilage invasion

	Radiologist	LR	LR-SVMSMOTE
AUC(95%CI)	0.721(0.663–0.774)	0.876(0.830–0.913)	0.905(0.863–0.937)
Sensitivity (%)	74.4	80.2	80.2
Specificity (%)	69.8	83.8	88.3
Accuracy (%)	71.3	82.6	85.7
Precision(%)	0.542	0.704	0.767
F1-score	0.627	0.750	0.784
Kappa	0.404	0.618	0.677
MCC	0.417	0.621	0.677
*P* Value		< 0.001^a^	< 0.001^a^

*LR* Logistic regression, *LR-SVMSMOTE* Logistic regression - support vector machine-based synthetic minority oversampling, *CI* Confidence Interval, *MCC* Matthews Correlation Coefficient

^a^Delong test for differences in AUC compared to radiologist assessment

## Discussion

Our study analyzed CT-radiomics features for the prediction of thyroid cartilage invasion from LHSCC and preliminarily established different predictive models with machine learning. In our study, the LR-SVMSMOTE and LR models showed relatively higher AUC (0.905 and 0.876, respectively) than assessment by the radiologist (0.721) in the prediction of thyroid cartilage invasion from LHSCC. The results demonstrated that CT-based radiomics features have great potential to act as noninvasive imaging markers for accurate prediction of thyroid cartilage invasion from LHSCC with a satisfactory predictive performance.

The majority of laryngeal cartilage ossifies and calcifies with aging. However, the process of ossification has great variability, especially in the thyroid cartilage [11]. Therefore, normal adult thyroid cartilage can be classified into three types: (1) no ossification, (2) cortical ossification, and (3) high fatty content in the medullary cavity of ossified cartilage [11, 34]. Sclerosis was identified as one of the criteria for thyroid cartilage invasion in a previous study [28]. Thus, asymmetric ossification of normal thyroid cartilage can be misdiagnosed as thyroid cartilage invasion. Moreover, the CT values of nonossified hyaline cartilage are similar to those of tumors [11, 12, 34], making it difficult to assess thyroid cartilage invasion with CT. On MRI, the differentiation of peritumoral inflammatory changes and thyroid cartilage invasion remains challenging and the specificity is low (around 65%) [13, 35]. Additionally, because of its longer imaging time, the quality of MR images can be degraded by swallowing or breathing movements. Compared with conventional methods, radiomics can be used for quantitative analysis of tumors, excavating the valuable information in CT images for patients with and without thyroid cartilage invasion and making diagnosis more accurate.

Clinical research using radiomics can be divided into five steps: (1) Data collection: targeted collection for a specific clinical question; (2) ROI segmentation: delineation of the target area in the images; (3) Feature selection: high-throughput extraction of lesion features; (4) Feature reduction: selection of features with high reliability from the feature set for model training to improve the generalization ability of the model; and (5) Model establishment [15, 36, 37].

Segmentation is one of the most important issues in radiomics. A study suggested that three-dimensional analysis may achieve better predictive performance than two-dimensional analysis for kidney masses [38]. In our study, all slices of the tumor were manually delineated on CE-CT images into 3-mm thick reconstructed sections. This is a laborious and time-consuming process. Whether better results could be achieved by including all slices in the analysis rather than using the maximum cross sectional area in LHSCC is unknown. It should be noted that delineating the VOIs on CT can be challenging and the result may not have been particularly accurate. The reason is that the contrast between lesions and normal structures is often low in CT images. In spite of this limitation, the interobserver agreement in this study was good. Compared with CT, the tumor boundary is often more clearly observed on MRI. Hence MRI-based radiomics features in LHSCC may provide better predictive performance compared to CT. Our study focused only on the aggressiveness of the tumor itself and the thyroid cartilage was not examined. Perhaps segmentation of the thyroid cartilage can be performed in the future to achieve better results.

In our study at the beginning of the model establishment process, 1029 features were extracted to reduce deviations in the model resulting from a lack of important features. However, the optimal feature subset with the strongest correlation to thyroid cartilage invasion had to be determined during the modeling process, that is, feature selection was necessary to improve the accuracy of prediction for establishment of the model. The LASSO method is an estimation method that can achieve the reduction of feature sets and can analyze large sets of radiomics features with a relatively small sample size [37]. Twenty-two optimal subsets of 1029 radiomics features were found to distinguish thyroid cartilage invasion from non-invasion in LHSCC by using the LASSO method in our study. Of the 22 optimal feature subsets, the top three ranked features related to thyroid cartilage were “GrayLevelNonUniformity”(GLNU), “LeastAxis”, and “ShortRunHighGrayLevelEmphasis” (SRHGLE). GLNU is a textural feature derived from GLSZM. It quantifies the gray-level intensity values in the VOI. A higher value indicates more heterogeneity in the intensity values [29, 37]. SRHGLE is a textural feature calculated from GLRLM. It measures the joint distribution of shorter run lengths with higher gray-level values [29, 37]. The values of GLNU and SRHGLE in the thyroid cartilage invasion group were higher than those in the non-invasive thyroid cartilage group. It is likely that the two parameters reflect the spatial heterogeneity of the tumors. LeastAxis is a shape features that represents smallest axis length for the ROI-enclosing ellipsoid and has been proven proved be related to tumor invasiveness [20]. The thyroid cartilage invasion group had the larger leastaxis value in the current study. A predictive model was constructed using the LR classifier, which greatly improved the sensitivity and accuracy without sacrificing specificity. The LR classifier is the most popular supervised classifier in radiomics and is suitable for small sample and two-classification algorithms. It has also been successfully used in model construction for other tumors [20, 30, 37].

To address the imbalance in the dataset used in this study, the SVMSMOTE method was adopted to resample the thyroid cartilage invasion group such that the sample size for the group equaled that of the group without thyroid cartilage invasion. The SVMSMOTE method can alleviate the problem of overfitting without losing valuable information [22, 27]. The classifier of LR with SVMSMOTE can obtain a more optimal feature set after dimension reduction. “LeastAxis”, “ZoneEntropy” (ZE), and GLUN were the top three most important features in the results. ZE represents a textural feature that originates from GLSZM. ZE mainly reflects the textural complexity of lesion (the higher the ZE value, the more complex the texture). Compared with the thyroid cartilage noninvasive group, the invasive group had higher ZE value (consistent with faster growth and greater tumor heterogeneity) [29]. The LR-SVMSMOTE model had better AUC, specificity, and accuracy than the LR model. Further, the accuracy of the two different radiomics models (LR-SVMSMOTE and LR) was superior to that of the less experienced radiologist. Thyroid cartilage invasion can be quantitatively diagnosed without relying on the experience of radiologists and has the potential to help with the diagnosis of radiologists. Quantitative prediction using radiomics for diseases not only avoids potential inaccuracy from observers subjectively interpreting the imaging findings, but also integrates imaging features that are difficult to distinguish with the naked eyes [36, 39].Clinicians can utilize individualized therapy to improve the 5-year survival rate and quality of life for patients with LHSCC [8].

Our study had several limitations. First, we manually delineated all of the slices of the lesion, which was time-consuming. A CT-based semi-automatic segmentation method was recently used for radiomics analysis of lung tumors [40] and a fully automatic segmentation approach using MRI has been performed for brain cancer [41]. A reliable and stable automatic segmentation method needs to be developed for LHSCC in the future so as to greatly reduce the burden of researchers. Second, only venous phase CE-CT images were segmented and the related radiomics features were extracted. The advantages and disadvantages of non-enhanced and arterial phase CE-CT images were not compared. Thus, more intensive research will be needed in the future. Third, our CT scans were performed with three different scanners and the different scanning parameters might have affected the results. However, we used the KW nonparametric test to remove radiomics features with statistical differences among the three machines. In addition, our conventional radiology assessment was conducted by two junior radiologists, not senior radiologists. These two junior radiologists had interpreted a large number of CT images for LHSCC in the Head and Neck Specialist Hospital for 3 years. Nevertheless, the interpretations of senior radiologists still need to be compared with assessments using radiomics to determine the similarities and whether additional information is obtained using the radiomics approach. Furthermore, our study adopted cross-validation, which may not avoid the overfitting risk, a held-out test set and external validation are needed to further validate the performance of the models.

## Conclusions

In conclusion, the present study showed that models based on CT radiomic features had higher AUCs than radiologist assessment in the prediction of thyroid cartilage invasion from LHSCC. The classifier comprised of LR with SVMSMOTE was able to identify the presence of thyroid cartilage invasion and the AUC reached 0.905 in this study. This technique provides a new noninvasive method for preoperative prediction of thyroid cartilage invasion from LHSCC with satisfactory predictive performance. However, it should be clear that this is a proof of concept study and the results remains to be proven, with external validation and prospective clinical studies.

## Supplementary Information

**Additional file 1.** Details of the radiomics features are shown in Supplementary S1.

## Data Availability

The datasets used or analysed during the current study are available from the corresponding author on reasonable request.

## Abbreviations

LHSCC: Laryngeal and hypopharyngeal squamous cell carcinoma
CT: Computed tomography
LASSO: The least absolute shrinkage and selection operator
LR: Logistic regression
SVMSMOTE: The support vector machine-based synthetic minority oversampling technique
ROC: Receiver operating characteristic
MRI: Magnetic resonance imaging
ROI: Region of interest
MD-CT: Multi-detector row CT
VOI: Volume of interest
ICC: Inter-class correlation coefficient
GLCM: Gray level co-occurrence matrix
GLRLM: Gray level run length matrix
GLSZM: Gray level size zone matrix
AUC: Area under the curve
GLNU: Gray level non-uniformity
SRHGLE: Short run high gray level emphasis
ZE: Zone entropy
