# Supplementary material for: CT-based radiomics features in the prediction of thyroid cartilage invasion from laryngeal and hypopharyngeal squamous cell carcinoma
Source: Cancer Imaging. 2020 Nov 11;20:81. doi: 10.1186/s40644-020-00359-2 (PMC7661189; doi:10.1186/s40644-020-00359-2)
Supplement: Supplementary file 1 — Additional file 1. Details of the radiomics features are shown in Supplementary S1. [file 40644_2020_359_MOESM1_ESM.docx]

**Supplementary S1**

**Radiomic Features**

A total of 1029 radiomics features were extracted using the Radcloud platform (<https://mics.radcloud.cn>, Huiying Medical Technology Co., Ltd) based on the VOI for each patient determined from the CE-CT original images and filtered images. These comprised three groups of features, namely, intensity histogram features, shape and size features, and texture features [1].

In particular, shape and size features were often used to describe the geometric shape and size of the tumor, and were only calculated from the original images not the filtered images. Additionally, texture features were further divided into three subgroups named gray level co-occurrence matrix (GLCM), gray level run length matrix (GLRLM), and gray-level size zone matrix (GLSZM) [1].

The filters consisted of an exponential filter, square filter, square root filter, logarithmic filter, and wavelet decomposition. The wavelet decomposition included eight low-pass and high-pass filtered three-dimensional wavelet transforms: wavelet-LHL, wavelet-LHH, wavelet-HLL, wavelet-LLH, wavelet-HLH, wavelet-HHH, wavelet-HHL, and wavelet-LLL [2]. The schematic diagram of wavelet decomposition is shown in Figure S1 [3].

The features of the intensity histogram feature group, shape and size feature group, GLCM subgroup, GLRLM subgroup, and GLSZM subgroup are listed in Table S1. In conclusion, the number of features (1029) = shape and size features (15) + [intensity histogram features (19) + GLCM (27) + GLRLM (16) + GLSZM (16)] × the number of images (13)


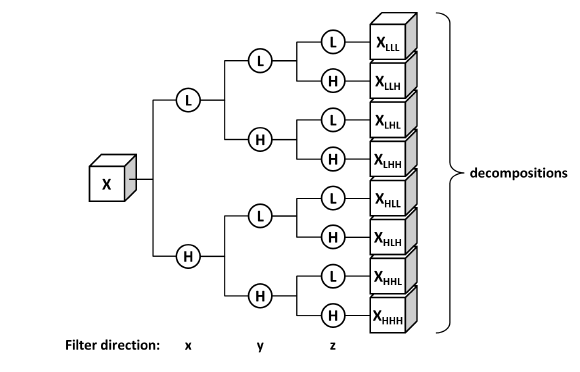


**Figure S1.**

Schematic of three-dimensional wavelet transform applied to each image. The original X image was decomposed into 8 decompositions: X_LHL_, X_LHH_, X_HLL_, X_LLH_, X_HLH_, X_HHH_, X_HHL_ and X_LLL_.

**Table S1.** The lists of radiomic features

| **Group/Subgroup** | **Radiomic feature** |
| --- | --- |
| **shape and size features (15)** | Volume, Surface Area, Surface Area to Volume Ratio, Sphericity, Compactness1, Compactness2, Spherical Disproportion, Maximum 3D Diameter, Maximum 2D Diameter Column, Maximum 2D Diameter Row, Major Axis, Minor Axis, Least Axis, Elongation, Flatness |
| **intensity histogram features (19)** | Energy, Total Energy, Entropy, Minimum, Maximum, 10Percentile, 90Percentile, Mean, Median, Interquartile Range, Range, Mean Absolute Deviation (MAD), Robust Mean Absolute Deviation (rMAD), Root Mean Squared (RMS), Standard Deviation, Skewness, Kurtosis, Variance, Uniformity |
| **GLCM (27)** | Auto-correlation, Average Intensity, Cluster Prominence, Cluster Shade, Cluster Tendency, Contrast, Correlation, Difference Average, Difference Entropy, Difference Variance, Energy, Entropy, Informal Measure of Correlation1 (IMC1), Informal Measure of Correlation2 (IMC2), Inverse Difference Moment (IDM), Inverse Difference Moment Normalized(IDMN), Inverse Difference(ID), Inverse Difference Normalized (IDN), Inverse Variance, Maximum Probability, Sum Average, Sum Entropy, Sum of Squares, Dissimilarity, Homogeneity1, Homogeneity2, Sum Variance |
| **GLRLM (16)** | Short Run Emphasis (SRE), Long Run Emphasis (LRE), Gray Level Non-Uniformity (GLN), Gray Level Non-Uniformity Normalized (GLNN), Run Length Non-Uniformity (RLN), Run Length Non-Uniformity Normalized (RLNN), Run Percentage (RP), Gray Level Variance (GLV), Run Variance (RV), Run Entropy (RE), Low Gray Level Run Emphasis (LGLRE), High Gray Level Run Emphasis (HGLRE), Short Run Low Gray Level Emphasis (SRLGLE), Short Run High Gray Level Emphasis (SRHGLE), Long Run Low Gray Level Emphasis (LRLGLE), Long Run High Gray Level Emphasis (LRHGLE) |
| **GLSZM (16)** | Small Area Emphasis (SAE), Large Area Emphasis (LAE), Gray Level Non-Uniformity (GLN), Gray Level Non-Normalized (GLNN), Size Zone Non-Uniformity (SZN), Size Zone Non-Uniformity Normalized (SZNN), Zone Percentage (ZP), Gray Level Variance (GLV), Zone Variance (ZV), Zone Entropy (ZE), Low Gray Level Zone Emphasis (LGLZE), High Gray Level Zone Emphasis (HGLZE), Small Area Low Gray Level Emphasis (SALGLE), Small Area High Gray Level Emphasis (SAHGLE), Large Area Low Gray Level Emphasis (LALGLE), Large Area High Gray Level Emphasis(LAHGLE) |

***Filters***

- **Exponential Filter**

$$f(x)=e^{cx},c=\frac{\log(\max(\left|x\right|))}{\max(\left|x\right|)}$$

- **Square Filter**

$$f(x)=(cx)^{2},c=\frac{1}{\sqrt{\max(\left|x\right|)}}$$

- **Square root Filter**

$$f(x)=,\left\{\begin{matrix} \sqrt{cx} & \begin{matrix} for & x\geq0 \end{matrix} \\ -\sqrt{-cx} & \begin{matrix} for & x<0 \end{matrix} \end{matrix},\right.c=\max(\left|x\right|)$$

- **Logarithm Filter**

$$f(x)=,\left\{\begin{matrix} \text{clog}(x+1) & \begin{matrix} for & x\geq0 \end{matrix} \\ -c\log(-x+1) & \begin{matrix} for & x<0 \end{matrix} \end{matrix},\right.c=\frac{\max(\left|x\right|)}{\log(\max(\left|x\right|)+1)}$$

Where $x$ and $f(x)$ are the original and filtered image for all the above filters, respectively.

***Intensity histogram features***

Notations:

$X$is an image of $N$voxels included in the VOI

$Pi$is the first order histogram with $N_{l}$discrete intensity levels, in which $N_{l}$is the number of non-zero bins,

$pi$is the normalized first order histogram and equal to $\frac{P_{i}}{\sum P_{i}}$ (This definition is the same for the following sections)

- **Energy**

$$energy=\sum_{i=1}^{N} (X(i)+c)^{2}$$

Here, c is optional value, which shifts the intensities to prevent negative values in $X$. This ensures that voxels with the lowest gray values contribute the least to Energy, instead of voxels with gray level intensity closest to 0.

- **Total Energy**

$$total\text{ }energy=V_{voxel}\sum_{i=1}^{N} (X(i)+c)^{2}$$

- **Entropy**

$$entropy=-\sum_{i=1}^{N_{l}} p(i)\log_{2}(p(i)+\varepsilon)$$

Here, $\varepsilon$ is an arbitrarily small positive number ($\approx2.2\times10^{\text{-}16}$)

- **Minimum**

$$\text{minimum }=\min(X)$$

- **Maximum**

$$\text{maximum }=\max(X)$$

- **10Percentile**

$$\text{The} 10^{\text{th}} \text{percentile} \text{of} X$$

- **90Percentile**

$$\text{The} 90^{\text{th}} \text{percentile} \text{of} X$$

- **Mean**

$$mean=\frac{1}{N}\sum_{i=1}^{N} X(i)$$

- **Median**

$$\text{The median gray level intensity within the VOI}$$

- **Interquartile Range**

$$\text{interquartile} \text{range}=P_{75}-P_{25}$$

- **Range**

$$\text{range }=\max(X)\text{-min}(X)$$

- **Mean Absolute Deviation (MAD)**

$$MAD=\frac{1}{N}\sum_{i=1}^{N} \left|X(i)-\overline{X}\right|$$

- **Robust Mean Absolute Deviation (rMAD)**

$$\text{r}MAD=\frac{1}{N_{10-90}}\sum_{i=1}^{N_{10-90}} \left|X_{10-90}(i)-\overline{X}_{10-90}\right|$$

- **Root Mean Squared (RMS)**

$$RMS=\sqrt{\frac{1}{N}\sum_{i=1}^{N} \left(X(i)+\text{c}\right)^{2}}$$

- **Standard Deviation**

$$\text{standard} \text{deviation}=\sqrt{\frac{1}{N}\sum_{i=1}^{N} \left(X(i)-\overline{X}\right)^{2}}$$

- **Skewness**

$$\text{skewness}=\frac{\mu_{3}}{\sigma^{3}}=\frac{\frac{1}{N}\sum_{i=1}^{N} (X(i)-\overline{X})^{3}}{\left(\sqrt{\frac{1}{N}\sum_{i=1}^{N} (X(i)-\overline{X})^{2}}\right)^{3}}$$

- **Kurtosis**

$$\text{kurtosis}=\frac{\mu_{4}}{\sigma^{4}}=\frac{\frac{1}{N}\sum_{i=1}^{N} (X(i)-\overline{X})^{4}}{\left(\sqrt{\frac{1}{N}\sum_{i=1}^{N} (X(i)-\overline{X})^{2}}\right)^{4}}$$

- **Variance**

$$\text{variance}=\frac{1}{N}\sum_{i=1}^{N} \left(X(i)-\overline{X}\right)^{2}$$

- **Uniformity**

$$\text{uniformity}=\sum_{i=1}^{N_{l}} p(i)^{2}$$

***Shape and size features***

- **Volume**

$$V=\sum_{i=1}^{N} V_{i}$$

- **Surface Area**

$$A=\sum_{i=1}^{N} \frac{1}{2}\left|a_{i}b_{i}\times a_{i}c_{i}\right|$$

$a_{i}b_{i} \text{and} a_{i}c_{i}$are the adges of the i^th^ triangle formed by points a_i_, b_i_ and c_i_

- **Surface Area to Volume Ratio**

$$\text{surface to volume ratio=}\frac{\text{A}}{\text{V}}$$

- **Sphericity**

- **Compactness1**

$$compactness1=\frac{V}{\sqrt{\pi A^{3}}}$$

- **Compactness2**

$$compactness2=36\pi\frac{V^{2}}{A^{3}}$$

- **Spherical Disproportion**

- **Maximum 3D Diameter**

The maximum three-dimensional diameter is measured as the largest pairwise Euclidean distance, between voxels on the surface of the tumor volume.

- **Maximum 2D Diameter Column**

The maximum 2D diameter (Column) is defined as the largest pairwise Euclidean distance between surface voxels in the coronal plane.

- **Maximum 2D Diameter Row**

The maximum 2D diameter (Row) is defined as the largest pairwise Euclidean distance between surface voxels in the sagittal plane.

- **Major Axis**

$$\text{major axis=4}\sqrt{\lambda_{\text{major}}}$$

- **Minor Axis**

$$\text{minor axis=4}\sqrt{\lambda_{\text{minor}}}$$

- **Least Axis**

$$\text{least axis=4}\sqrt{\lambda_{\text{least}}}$$

- **Elongation**

$$\text{elongation=}\sqrt{\frac{\lambda_{\text{minor}}}{\lambda_{\text{major}}}}$$

- **Flatness**

$$\text{flatness=}\sqrt{\frac{\lambda_{\text{least}}}{\lambda_{\text{major}}}}$$

Here, $\lambda_{\text{major}}, \lambda_{\text{minor}} and \lambda_{\text{least}}$ are the lengths of the largest, second largest and smallest principal component axes.

***GLCM features***

A Gray Level Co-occurrence Matrix (GLCM) of size $N\times N$ describes the second-order joint probability function of an image region constrained by the mask [1,3,4]. In our study, GLCM was the symmetrical matrix which means co-occurrences should be counted in two directions in each angle, and the distance between two voxels was set as 1 with 13 kinds of angles of 2 neighbors in three-dimensional images. The values of feature were calculated on the GLCM for each angle individually, and then took the average.

Notations:

$P(i,j)$is the co-occurrence matrix for $\delta$(distance) and $\alpha$(angle)

$p(i,j)$is the normalized co-occurrence matrix equal to $\frac{P_{i}}{\sum P_{i}}$

$N_{\text{g}}$ is the number of discrete intensity levels in the image

$p_{x}(i)=\sum_{j=1}^{N_{g}} p(i,j)$is the marginal row probability

$p_{y}\left( j \right)=\sum_{i=1}^{N_{g}} p\left( i,j \right)$is the marginal column probability

$\mu_{x}=\sum_{i=1}^{N_{g}} \sum_{j=1}^{N_{g}} p(i,j)i$is the mean gray level intensity of $p_{x}$

$\mu_{\text{y}}=\sum_{i=1}^{N_{g}} \sum_{j=1}^{N_{g}} p(i,j)j$is the mean gray level intensity of $p_{\text{y}}$

$\sigma_{\text{x}}$ is the standard deviation of $p_{x}$

$\sigma_{\text{y}}$ is the standard deviation of $p_{\text{y}}$

$$p_{x+y}(k)=\sum_{i=1}^{N_{g}} \sum_{j=1}^{N_{g}} p(i,j), where i+j=k$$

$$p_{x\text{-}y}(k)=\sum_{i=1}^{N_{g}} \sum_{j=1}^{N_{g}} P(i,j), where \left|i\text{-}j\right|=k$$

$HX=-\sum_{i=1}^{N_{g}} \sum_{j=1}^{N_{g}} p_{x}(i)\log_{2}(p_{x}(i)+\varepsilon)$is the entropy of $p_{x}$

$HY=-\sum_{i=1}^{N_{g}} \sum_{j=1}^{N_{g}} p_{y}(i)\log_{2}(p_{y}(i)+\varepsilon)$is the entropy of $p_{\text{y}}$

$HXY=-\sum_{i=1}^{N_{g}} \sum_{j=1}^{N_{g}} p(i,j)\log_{2}(p(i,j)+\varepsilon)$is the entropy of $p(i,j)$

$$HXY1=-\sum_{i=1}^{N_{g}} \sum_{j=1}^{N_{g}} p(i,j)\log_{2}(p_{x}(i)p_{y}(i)+\varepsilon)$$

$$HXY2=-\sum_{i=1}^{N_{g}} \sum_{j=1}^{N_{g}} p_{x}(i)p_{y}(i)\log_{2}(p_{x}(i)p_{y}(i)+\varepsilon)$$

- **Auto-correlation**

$$auto-correlation=\sum_{i=1}^{N_{g}} \sum_{j=1}^{N_{g}} p(i,j)ij$$

- **Average Intensity**

$$\text{average} \text{intensity}=\mu_{x}=\sum_{i=1}^{N_{g}} \sum_{j=1}^{N_{g}} p(i,j)i$$

- **Cluster Prominence**

$$\text{cluster prominence}=\sum_{i=1}^{N_{g}} \sum_{j=1}^{N_{g}} (i+j-\mu_{x}-\mu_{y})^{4}p(i,j)$$

- **Cluster Shade**

$$\text{cluster shade}=\sum_{i=1}^{N_{g}} \sum_{j=1}^{N_{g}} (i+j-\mu_{x}-\mu_{y})^{3}p(i,j)$$

- **Cluster Tendency**

$$\text{cluster tendency}=\sum_{i=1}^{N_{g}} \sum_{j=1}^{N_{g}} (i+j-\mu_{x}-\mu_{y})^{2}p(i,j)$$

- **Contrast**

$$\text{contrast}=\sum_{i=1}^{N_{g}} \sum_{j=1}^{N_{g}} (i-j)^{2}p(i,j)$$

- **Correlation**

$$correlation=\frac{\sum_{i=1}^{N_{g}} \sum_{j=1}^{N_{g}} p(i,j)ij-\mu_{x}\mu_{y}}{\sigma_{x}(i)\sigma_{y}(j)}$$

- **Difference Average**

$$difference average=\sum_{k=0}^{N_{g}-1} kp_{x-y}(k)$$

- **Difference Entropy**

$$difference entropy=\sum_{k=0}^{N_{g}-1} p_{x-y}(k)\log_{2}(p_{x-y}(k)+\varepsilon)$$

- **Difference Variance**

$$\text{difference} \text{varinance}=\sum_{k=0}^{N_{g}-1} (k-DA)^{2}p_{x-y}(k)$$

- **Energy**

$$energy=\sum_{i=1}^{N_{g}} \sum_{j=1}^{N_{g}} (p(i-j))^{2}$$

- **Entropy**

$$entropy=\text{-}\sum_{i=1}^{N_{g}} \sum_{j=1}^{N_{g}} p(i,j)\log_{2}(p(i,j)+\varepsilon)$$

- **Informal Measure of Correlation1 (IMC1)**

$$IMC1=\frac{HXY-HXY1}{\max\{HX,HY\}}$$

- **Informal Measure of Correlation2 (IMC2)**

$$IMC2=\sqrt{\text{1-e}^{-2(HXY2-HXY)}}$$

- **Inverse Difference Moment (IDM)**

$$\text{I}DM=\sum_{i=1}^{N_{g}} \sum_{j=1}^{N_{g}} \frac{p(i,j)}{1+\left|i-j\right|^{2}}$$

- **Inverse Difference Moment Normalized (IDMN)**

$$\text{I}DMN=\sum_{i=1}^{N_{g}} \sum_{j=1}^{N_{g}} \frac{p(i,j)}{1+\left(\frac{\left|i-j\right|^{2}}{N_{\text{g}}^{2}}\right)}$$

- **Inverse Difference (ID)**

$$\text{ID}=\sum_{i=1}^{N_{g}} \sum_{j=1}^{N_{g}} \frac{p(i,j)}{1+\left|i-j\right|}$$

- **Inverse Difference Normalized (IDN)**

$$\text{I}DN=\sum_{i=1}^{N_{g}} \sum_{j=1}^{N_{g}} \frac{p(i,j)}{1+\left(\frac{\left|i-j\right|}{N_{g}}\right)}$$

- **Inverse Variance**

$$\text{inverse} \text{variance}=\sum_{i=1}^{N_{g}} \sum_{j=1}^{N_{g}} \frac{p(i,j)}{\left|i-j\right|^{2}},i\neq j$$

- **Maximum Probability**

$$\text{maximum probability}=\max(p(i,j))$$

- **Sum Average**

$$sum average=\sum_{k=2}^{2N_{g}} p_{x+y}(k)k$$

- **Sum Entropy**

$$sum entropy=\sum_{k=2}^{2N_{g}} p_{x+y}(k)\log_{2}(p_{x+y}(k)+\varepsilon)$$

- **Sum of Squares**

$$sum squares=\sum_{i=1}^{N_{g}} \sum_{j=1}^{N_{g}} (i-\mu_{x})^{2}p(i,j)$$

- **Dissimilarity**

$$dissimilarity=\sum_{i=1}^{N_{g}} \sum_{j=1}^{N_{g}} \left|i-j\right|p(i,j)$$

- **Homogeneity1**

$$\text{homogeneity} 1=\sum_{i=1}^{N_{g}} \sum_{j=1}^{N_{g}} \frac{p(i,j)}{1+\left|i-j\right|}$$

- **Homogeneity2**

$$\text{homogeneity} 2=\sum_{i=1}^{N_{g}} \sum_{j=1}^{N_{g}} \frac{p(i,j)}{1+\left|i-j\right|^{2}}$$

- **Sum Variance**

$$\text{sum} \text{variance}=\sum_{k=2}^{2N_{g}} (k-SA)^{2}p_{x+y}(k)$$

***GLRLM features***

A Gray Level Run Length Matrix (GLRLM) quantifies gray level runs, which are defined as the length in number of pixels, of consecutive pixels that have the same gray level value [1,3,5].

Notations:

$P(i,j|\theta)$is the run length matrix for $\theta$(direction)

$p(i,j\text{|}\theta)$is the normalized run length matrix, defined as $p\left( i,j|\theta\right)=\frac{P\left( i,j|\theta\right)}{N_{z}}$

$N_{\text{g}}$ is the number of discrete intensity levels in the image

$N_{\text{r}}$ is the number of discrete run lengths in the image

$N_{\text{p}}$ is the number of voxels in the image

$N_{\text{z}}(\theta)$ is the number of runs in the image along angle $\theta$, $N_{\text{z}}(\theta)=\sum_{i=1}^{N_{\text{g}}} \sum_{j=1}^{N_{r}} P(i,j|\theta)$

- **Short Run Emphasis (SRE)**

$$SRE=\frac{\sum_{i=1}^{N_{\text{g}}} \sum_{j=1}^{N_{r}} \frac{P(i,j|\theta)}{j^{2}}}{N_{z}(\theta)}$$

- **Long Run Emphasis (LRE)**

$$LRE=\frac{\sum_{i=1}^{N_{\text{g}}} \sum_{j=1}^{N_{r}} P(i,j|\theta)j^{2}}{N_{z}(\theta)}$$

- **Gray Level Non-Uniformity (GLN)**

$$GLN=\frac{\sum_{i=1}^{N_{\text{g}}} (\sum_{\text{j}=1}^{N_{\text{r}}} P(i,j|\theta))^{2}}{N_{\text{z}}(\theta)}$$

- **Gray Level Non-Uniformity Normalized (GLNN)**

$$GLN=\frac{\sum_{i=1}^{N_{\text{g}}} (\sum_{\text{j}=1}^{N_{\text{r}}} P(i,j|\theta))^{2}}{N_{\text{z}}(\theta)^{2}}$$

- **Run Length Non-Uniformity (RLN)**

$$RLN=\frac{\sum_{j=1}^{N_{\text{r}}} (\sum_{\text{i}=1}^{N_{\text{g}}} P(i,j|\theta))^{2}}{N_{\text{z}}(\theta)}$$

- **Run Length Non-Uniformity Normalized (RLNN)**

$$RLNN=\frac{\sum_{j=1}^{N_{\text{r}}} (\sum_{\text{i}=1}^{N_{\text{g}}} P(i,j|\theta))^{2}}{N_{\text{z}}(\theta)^{2}}$$

- **Run Percentage (RP)**

$$RP=\frac{N_{z}(\theta)}{N_{p}}$$

- **Gray Level Variance (GLV)**

$$GLV=\sum_{i=1}^{N_{\text{g}}} \sum_{j=1}^{N_{r}} p(i,j|\theta)(i-\mu)^{2},\mu=\sum_{i=1}^{N_{\text{g}}} \sum_{j=1}^{N_{r}} p(i,j|\theta)i$$

- **Run Variance (RV)**

$$RV=\sum_{i=1}^{N_{\text{g}}} \sum_{j=1}^{N_{r}} p(i,j|\theta)(j-\mu)^{2},\mu=\sum_{i=1}^{N_{\text{g}}} \sum_{j=1}^{N_{r}} p(i,j|\theta)j$$

- **Run Entropy (RE)**

$$RE=\text{-}\sum_{i=1}^{N_{g}} \sum_{j=1}^{N_{\text{r}}} p(i,j|\theta)\log_{2}(p(i,j|\theta)+\varepsilon)$$

- **Low Gray Level Run Emphasis (LGLRE)**

$$LGLRE=\frac{\sum_{i=1}^{N_{\text{g}}} \sum_{j=1}^{N_{r}} \frac{P(i,j|\theta)}{i^{2}}}{N_{z}(\theta)}$$

- **High Gray Level Run Emphasis (HGLRE)**

$$HGLRE=\frac{\sum_{i=1}^{N_{\text{g}}} \sum_{j=1}^{N_{r}} P(i,j|\theta)i^{2}}{N_{z}(\theta)}$$

- **Short Run Low Gray Level Emphasis (SRLGLE)**

$$SRLGLE=\frac{\sum_{i=1}^{N_{\text{g}}} \sum_{j=1}^{N_{r}} \frac{P(i,j|\theta)}{i^{2}j^{2}}}{N_{z}(\theta)}$$

- **Short Run High Gray Level Emphasis (SRHGLE)**

$$SRHGLE=\frac{\sum_{i=1}^{N_{\text{g}}} \sum_{j=1}^{N_{r}} \frac{P(i,j|\theta)i^{2}}{j^{2}}}{N_{z}(\theta)}$$

- **Long Run Low Gray Level Emphasis (LRLGLE)**

$$LRLGLE=\frac{\sum_{i=1}^{N_{\text{g}}} \sum_{j=1}^{N_{r}} \frac{P(i,j|\theta)j^{2}}{i^{2}}}{N_{z}(\theta)}$$

- **Long Run High Gray Level Emphasis (LRHGLE)**

$$LRHGLE=\frac{\sum_{i=1}^{N_{\text{g}}} \sum_{j=1}^{N_{r}} P(i,j|\theta)i^{2}j^{2}}{N_{z}(\theta)}$$

***GLSZM features***

A Gray Level Size Zone (GLSZM) quantifies gray level zones in an image. A gray level zone is defined as a number of connected voxels that share the same gray level intensity [1,6].

Notations:

$N_{\text{g}}$ is the number of discrete intensity levels in the image

$N_{\text{s}}$ is the number of discrete zone sizes in the image

$N_{\text{p}}$ is the number of voxels in the image

$N_{\text{z}}$ is the number of zones in the image $N_{\text{z}}=\sum_{i=1}^{N_{\text{g}}} \sum_{j=1}^{N_{r}} P(i,j)$

$P(i,j)$is the size zone matrix

$p(i,j)$is the normalized size zone matrix, defined as $p\left( i,j \right)=\frac{P\left( i,j \right)}{N_{z}}$

- **Small Area Emphasis (SAE)**

$$SAE=\frac{\sum_{i=1}^{N_{\text{g}}} \sum_{j=1}^{N_{s}} \frac{P(i,j)}{j^{2}}}{N_{z}}$$

- **Large Area Emphasis (LAE)**

$$LAE=\frac{\sum_{i=1}^{N_{\text{g}}} \sum_{j=1}^{N_{s}} P(i,j)j^{2}}{N_{z}}$$

- **Gray Level Non-Uniformity (GLN)**

$$GLN=\frac{\sum_{i=1}^{N_{\text{g}}} (\sum_{\text{j}=1}^{N_{\text{s}}} P(i,j))^{2}}{N_{\text{z}}}$$

- **Gray Level Non-Uniformity Normalized (GLNN)**

$$GLN=\frac{\sum_{i=1}^{N_{\text{g}}} (\sum_{\text{j}=1}^{N_{\text{s}}} P(i,j))^{2}}{N_{\text{z}}^{2}}$$

- **Size Zone Non-Uniformity (SZN)**

$$SZN=\frac{\sum_{j=1}^{N_{\text{s}}} (\sum_{\text{i}=1}^{N_{\text{g}}} P(i,j))^{2}}{N_{\text{z}}}$$

- **Size Zone Non-Uniformity Normalized (SZNN)**

$$SZNN=\frac{\sum_{j=1}^{N_{\text{s}}} (\sum_{\text{i}=1}^{N_{\text{g}}} P(i,j))^{2}}{N_{\text{z}}^{2}}$$

- **Zone Percentage (ZP)**

$$ZP=\frac{N_{z}}{N_{p}}$$

- **Gray Level Variance (GLV)**

$$GLV=\sum_{i=1}^{N_{\text{g}}} \sum_{j=1}^{N_{\text{s}}} p(i,j)(i-\mu)^{2},\mu=\sum_{i=1}^{N_{\text{g}}} \sum_{j=1}^{N_{s}} p(i,j)i$$

- **Zone Variance (ZV)**

$$ZV=\sum_{i=1}^{N_{\text{g}}} \sum_{j=1}^{N_{\text{s}}} p(i,j)(j-\mu)^{2},\mu=\sum_{i=1}^{N_{\text{g}}} \sum_{j=1}^{N_{s}} p(i,j)j$$

- **Zone Entropy (ZE)**

$$ZE=\text{-}\sum_{i=1}^{N_{g}} \sum_{j=1}^{N_{\text{s}}} p(i,j)\log_{2}(p(i,j)+\varepsilon)$$

- **Low Gray Level Zone Emphasis (LGLZE)**

$$LGLZE=\frac{\sum_{i=1}^{N_{\text{g}}} \sum_{j=1}^{N_{\text{s}}} \frac{P(i,j)}{i^{2}}}{N_{z}}$$

- **High Gray Level Zone Emphasis (HGLZE),**

$$HGLZE=\frac{\sum_{i=1}^{N_{\text{g}}} \sum_{j=1}^{N_{\text{s}}} P(i,j)i^{2}}{N_{z}}$$

- **Small Area Low Gray Level Emphasis (SALGLE)**

$$SALGLE=\frac{\sum_{i=1}^{N_{\text{g}}} \sum_{j=1}^{N_{s}} \frac{P(i,j)}{i^{2}j^{2}}}{N_{z}}$$

- **Small Area High Gray Level Emphasis (SAHGLE)**

$$SAHGLE=\frac{\sum_{i=1}^{N_{\text{g}}} \sum_{j=1}^{N_{s}} \frac{P(i,j)i^{2}}{j^{2}}}{N_{z}}$$

- **Large Area Low Gray Level Emphasis (LALGLE),**

$$LALGLE=\frac{\sum_{i=1}^{N_{\text{g}}} \sum_{j=1}^{N_{\text{s}}} \frac{P(i,j)j^{2}}{i^{2}}}{N_{z}}$$

- **Large Area High Gray Level Emphasis(LAHGLE)**

$$LAHGLE=\frac{\sum_{i=1}^{N_{\text{g}}} \sum_{j=1}^{N_{s}} P(i,j)i^{2}j^{2}}{N_{z}}$$

**Supplementary References**

1. van Griethuysen JJM, Fedorov A, Parmar C et al. Computational radiomics system to decode the radiographic phenotype. Cancer Res. 2017;77(21):e104–7.

2. Lee G, Gommers R, Waselewski F et al. PyWavelets: A Python package for wavelet analysis. J Open Source Softw. 2019;4(36):1237.

3. Aerts HJWL, Velazquez ER, Leijenaar RTH et al. Decoding tumour phenotype by noninvasive imaging using a quantitative radiomics approach. Nat Commun 2014;5(1):4006-13.

4. Haralick R M, Shanmugam K, Dinstein I. Textural features for image classification. Stud Media Commun.1973,3(6):610-21.

5. Galloway MM. Texture analysis using gray level run lengths. Comput Graph Image Process.1975;4(2):172–9.

6. Thibault G, Fertil B, Navarro C et al. Texture indexes and gray level size zone matrix application to cell nuclei classification. In Pattern Recognition Inf Process. 2009;140-5.
